# Activation effects on the physical characteristics of T lymphocytes

**DOI:** 10.3389/fbioe.2023.1175570

**Published:** 2023-05-15

**Authors:** Richard E. Waugh, Elena Lomakina, Andrea Amitrano, Minsoo Kim

**Affiliations:** ^1^ Department of Biomedical Engineering, University of Rochester, Rochester, NY, United States; ^2^ Department of Pathology, University of Rochester Medical Center, Rochester, NY, United States; ^3^ Department of Microbiology and Immunology, David H. Smith Center for Vaccine Biology and Immunology, University of Rochester, Rochester, NY, United States

**Keywords:** T cells, mechanics, cell activation, micropipette, cell therapeutics

## Abstract

The deformability of leukocytes is relevant to a wide array of physiological and pathophysiological behaviors. The goal of this study is to provide a detailed, quantitative characterization of the mechanical properties of T cells and how those properties change with activation. We tested T cells and CD8^+^ cells isolated from peripheral blood samples of healthy donors either immediately (naïve population) or after 7 days of activation *in vitro*. Single-cell micropipette aspiration was used to test the mechanical properties. T cells exhibit the general characteristics of a highly viscous liquid drop with a cortical “surface” tension, *T*_*cort*_. The time course of each cell entry into the micropipette was measured at two different aspiration pressures to test for shear thinning behavior. The data were analyzed in the framework of an approximate mechanical model of the cell deformation to determine the cortical tension, the cell volume, the magnitude of the initial cell entry, the characteristic viscosity *μ*_*o*_, and the shear thinning coefficient, *b*. Activation generally caused increases in cellular resistance to deformation and a broadening of the distribution of cell properties. The cell volume increased substantially upon cell activation from ∼200 μm^3^ to ∼650 μm^3^. Naive and activated T cells had similar mean cortical tension (∼150 pN/μm). However, compared to naïve CD8^+^ cells, the cortical tension of activated CD8^+^ cells increased significantly to ∼250 pN/μm. Dynamic resistance of naive CD8^+^ T cells, as reflected in their characteristic viscosity, was ∼870 Pa and significantly increased to 1,180 Pa after *in vitro* activation. The magnitude of the instantaneous projection length as the cell enters the pipette (*L*_*init*_) was more than doubled for activated vs. naive cells. All cell types exhibited shear thinning behavior with coefficients *b* in the range 0.5–0.65. Increased cell size, cortical tension, and characteristic viscosity all point to increased resistance of activated T cells to passage through the microvasculature, likely contributing to cell trapping. The increased initial elastic response of cells after activation was unexpected and could point to instability in the cell that might contribute to spontaneous cell motility.

## 1 Introduction

The deformability of leukocytes is relevant to a wide array of physiological and pathophysiological behaviors. It likely plays a role in the distribution of leukocytes in the vasculature, as well as trapping of cells in the lung and other tissues during cell therapy or pathological inflammatory responses (Worthen et al., 1989). It is of particular interest to understand how the mechanical properties of leukocytes may change under different conditions. For example, in the case of T-cell immunotherapy, a dramatic change in cell deformability during *in vitro* activation could lead to differences in how cells interact with the endothelium in different tissues, as well as differences in cell distribution and trapping, leading to uneven delivery of subpopulations within infused cell cohorts. In this study we have examined the deformability of unfractionated primary T cells *versus* CD8^+^ T cells and tested the effects of cell activation on their mechanical behavior.

It has been accepted for some time that from a mechanical perspective, leukocytes exhibit the general behavior of a highly viscous liquid droplet (Evans and Kukan, 1984). The cell exhibits a contractile force (cortical tension) at its periphery, akin to a surface tension, that is responsible for drawing the cell into its spherical shape (Tran-Son-Tay et al., 1991). Like the constant surface tension of a liquid, the cortical tension of cells exhibits only a weak dependence on surface stretching (Needham and Hochmuth, 1992; Lam et al., 2009). Once the resistance to deformation due to the cortical tension is overcome, the cell flows continuously in response to an applied force. This basic liquid-droplet behavior was first documented in neutrophils (Evans and Yeung, 1989), and it has subsequently been shown to be applicable to a wide range of cell types, including lymphocytes (Perrault et al., 2004) and a number of cultured cell lines (Tsai et al., 1996; Lam et al., 2009).

While most theoretical descriptions of leukocyte deformation treat the cell as a Newtonian fluid, early experiments provided evidence that cells exhibit shear thinning (Tsai et al., 1993): at higher rates of deformation, the apparent cell viscosity decreases. This behavior has been characterized in terms of a power-law fluid model, in which the viscosity *μ* at a given shear rate (*D*_max_) is related to the characteristic viscosity *μ*_*o*_ in a power-law relationship (Tsai et al., 1993):

$$\mu =\mu_{o}{\left(D_{\max}\right)}^{-b}$$ (1)

The characteristic viscosity is the viscosity when the rate of deformation *D*_max_ has a value of 1.0 s^-1^, and the power-law coefficient *b* determines the degree to which the viscosity changes with changing rate of deformation. This model has successfully explained widely divergent values reported for the cell viscosity using different approaches for measurement (Tsai et al., 1993), and power-law behavior appears to apply broadly to cells in general (Fabry et al., 2001).

While the general description of the cell as a power-law fluid captures most aspects of the cell behavior, there is an important discrepancy between the predictions of the power-law model and observed cell behavior. It has been observed across multiple labs that when a leukocyte is aspirated into a micropipette, there is an initial rapid entry phase before the cell begins to follow the behavior predicted by liquid drop models. Generally, this has been attributed to an initial elastic response (Schmid-Schonbein et al., 1981; Needham and Hochmuth, 1990), and computationally, different work-arounds have been used to account for this response in calculating the apparent cell viscosity. Needham and Hochmuth (Needham and Hochmuth, 1990) essentially ignored the initial rapid entry and focused their estimations of viscosity on the quasi-linear portion of the cell entry time course. Dong et al. (1988) proposed a model of a Maxwell fluid, exhibiting an initial elastic response followed by creeping flow. (Herant et al., 2003) proposed that the initial rapid entry might be accounted for in a model in which the majority of the cellular resistance to deformation might reside in its cortex, and that the initial projection might reflect a degree of “slack” in the folded membrane surface that needs to be pulled taut before significant dissipation occurs. In the following sections, we use a simplified geometric model to evaluate cellular viscosity using a model in which the initial elastic response is modeled phenomenologically as an exponential approach to an “initial” projection length, and a shear-thinning, viscous response for the remainder of the cell entry.

## 2 Materials and methods

### 2.1 Cell preparation

Blood samples were obtained by venipuncture from healthy donors under an IRB approved protocol at the University of Rochester. T cells were isolated using EasySep Direct Human T Cell Isolation kit (STEMCELL Technologies, Cambridge, MA). CD8 positive (CD8^+^) cells were isolated using EasySep Human Naïve CD8^+^ T Cell Isolation Kit II (STEMCELL Technologies, Cambridge, MA). For activation, cells were cultured seven or 8 days in TecMACS medium (Myltenyi Biotech, Auburn, CA) supplemented with 3% human AB serum (Sigma, St. Louis, MO) and 200U/ml of recombinant human IL-2 (PeproTech, Cranbury, NJ) on plate bound CD3 and CD28 antibodies.

For mechanical testing experiments we used L-15 media (Lonza, Walkersville, MD), supplemented with 2 mg/ml of glucose and 4% FBS. Naïve cells were used for the experiment within 2 hours after isolation (4 hours after phlebotomy).

*Terminology.* Technically all T cells isolated from the peripheral blood by this method are not “naïve” although most of them are. For simplicity, we refer to both the total T cell population and the CD8^+^ sub population as “naïve” when they are freshly isolated from the blood, and “activated” after having undergone culture on plate bound CD3 and CD28 antibodies. “T cells” refers to the mixed population of T cells isolated from the blood.

### 2.2 Micropipette preparation and experiments

Micropipettes were made from glass capillary tubing (0.9 mm outside diameter x 0.2 mm wall thickness x 7 cm length; Friedrich & Dimmock Inc., Millville, NJ) using a vertical pipette puller (Model 730; David Kopf Instruments, Tujunga, CA) and a microforge consisting of a micromanipulator and a heated glass bead mounted on an inverted microscope.

**FIGURE 1:**
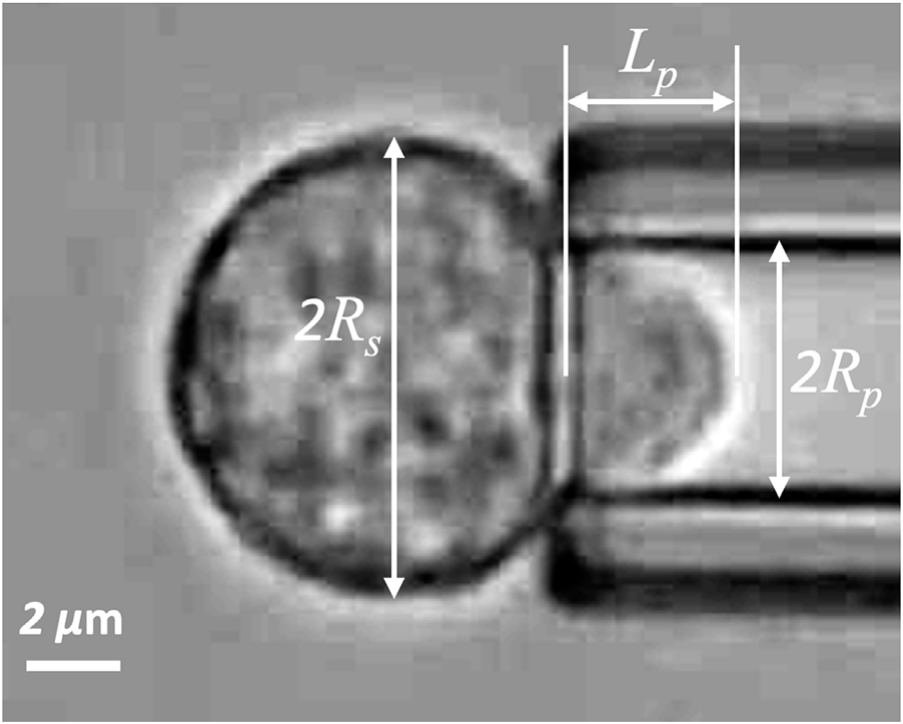
Naïve T-cell drawn into a micropipette. *R*_*s*_ is the radius of spherical portion of the cell outside the pipette, *R*_*p*_ is the pipette radius, and *L*_*p*_ is the length of the projection in the pipette.

All micropipette experiments were performed on the stage on the inverted microscope at room temperature using L-15 media supplemented with 2 mg/ml glucose and 4% fetal bovine serum (FBS). The micropipette was connected via a continuous water connection to a reservoir, the height of which could be adjusted using mechanical slide with a vernier (Unislide, Velmex, Bloomfield, NY). First, the cortical tension of the cell was estimated by measuring the critical pressure ( $P_{cr}$ ) (that is, the aspiration pressure at which the length of the cell projection neither increased nor decreased) for each individual cell. The cortical tension (*T*_*cort*_) was calculated using the following equation (Evans and Yeung, 1989):

$$T_{cort}=\frac{P_{cr}R_{p}R_{s}}{2\left(R_{s}-R_{p}\right)}$$ (2)

where $R_{p}$ is the pipette radius and $R_{s}$ is the radius of outer spherical segment of the cell at the critical pressure (Figure 1). After the cortical tension measurement, the cell was expelled and allowed to recover its spherical shape. Then a negative aspiration pressure ( $P_{1}$ ) was set by adjusting the height of the water reservoir and used to draw the cell into the pipette. The total length of the cell inside the micropipette was used to calculate the cell volume (see Supplementary Section S7) according to:

$$V=\pi {R_{p}}^{2}L_{tot}-2\pi {R_{p}}^{3}/3$$ (3)

After the cell was fully aspirated, the pressure was switched to positive to expel the cell into a chamber, and the cell was allowed to relax to its natural round shape (typically 1–2 min for naïve cells and 3–5 min for activated cells). Once the cell had recovered, a different negative pressure ( $P_{2}$ ) was applied and the cell was drawn into the pipette for a second time. Experiments were recorded, and the dimensions of the cell as a function of time during cell aspiration were measured using ImageJ (National Institute of Health, United States). Dimensions were calibrated using a recording of a stage micrometer. Pixel size was 0.07 μm. Resolution for time was limited by the video framing rate, 1/30 s. Repeated distance measurements showed a standard deviation of 0.1 um, making the 95% confidence interval for length measurements ± 0.2 μm (±3 pixels), approximately the limit of optical resolution for our microscope using monochromatic (488 nm) Koehler illumination.

### 2.3 Theoretical framework for data interpretation

#### 2.3.1 Solution for a power-law fluid

**FIGURE 2:**
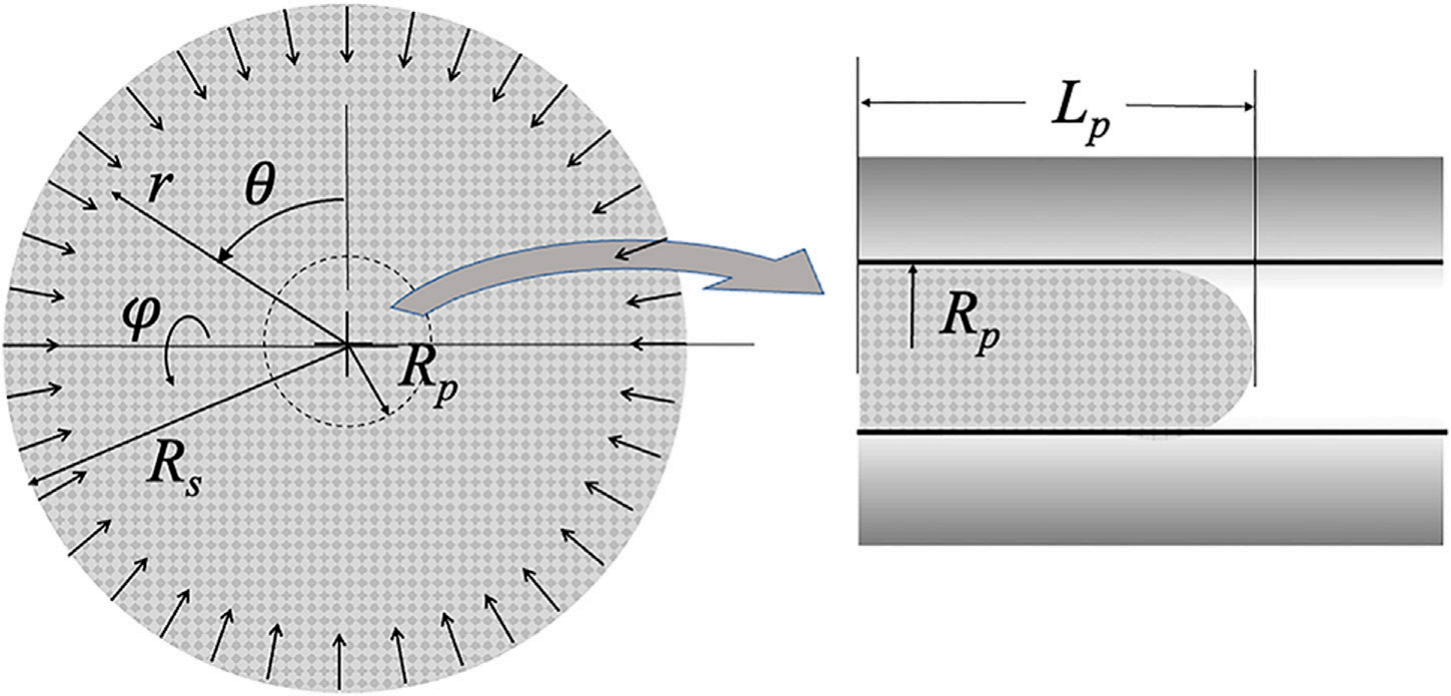
Schematic of the collapsing sphere model. Pipette radius *R*_*p*_ and projection length *L*_*p*_ are shown. The radius of the sphere *R*_*s*_ decreases with time as material crosses the *r* = *R*_*p*_ boundary and shows up in the micropipette. The origin of the spherical coordinates is the center of the sphere.

We use a geometric approximation of the cell as collapsing sphere (Figure 2). This approach was inspired by an approximation of the cell as a hemisphere being drawn into a cylinder first used by Needham and Hochmuth (Needham and Hochmuth, 1990). Both these approximations have the advantage that closed-form solutions can often be obtained to describe the cell deformation, vastly facilitating parameter estimation and model testing. The hemisphere approximation artificially increases the magnitude of deformation at late stages of entry because of the larger hemispherical radius needed to contain the cell volume. We circumvent this difficulty by recognizing that in the hemisphere model (see Supplementary Figure S2) all the dissipation occurs in the region between the outer radius *R*_*h*_ and the inner radius *R*_*p*_. Therefore, it is reasonable to replace the hemisphere with a sphere and solve the deformation as the outer radius *R*_*s*_ collapses to the inner radius *R*_*p*_. The rate of spherical collapse is set by equating the volume of material crossing the inner boundary (*r* = *R*_*p*_) with the volume of material entering the micropipette. Although this model probably underestimates the amount of deformation the cell undergoes as it enters the pipette, the model provides enormous computational advantages because of its symmetry, and enables us to more easily examine more complex constitutive relationships to evaluate and model cell behavior. Details are provided in the supplemental materials (Supplementary Sections S1–S3). Briefly, by setting the origin of a spherical coordinate system at the center of spherical portion of the cell, we observe that the deformation is invariant for both angular coordinates, *θ* and *φ*, making the entire system a function only of the radial coordinate *r*. We consider the flow field between the outer boundary of the cell, *r* = *R*_*s*_(*t*), and an inner boundary of radius *R*_*p*_ (the pipette radius). Neglecting inertial and gravitational terms, the equation of motion is (Bird et al., 1960) (p. 86)

∇p=∇∙τ (4)

where τ is the deviatoric stress tensor, and *p* is pressure. (Note that our sign convention for pressure is opposite that of the reference). The cortical tension *T*_*cort*_ enters the problem when we apply a force balance at the boundary of the cell. At the outer boundary (*r* = *R*_*s*_(*t*)):

$$-{\sigma_{rr}|}_{r=R_{s}}=p_{o}+\frac{2T_{cort}}{R_{s}}$$ (5a)

and at the inner boundary:

$$-{\sigma_{rr}|}_{r=R_{p}}=p_{p}+\frac{2T_{cort}}{R_{p}}$$ (5b)

where *p*_*p*_ is the pressure in the pipette lumen, and the total stress within the cell is *σ*_*rr*_ = -*p* + *τ*_*rr*_. (Note: consistent with the hemispherical model developed by Needham and Hochmuth (Needham and Hochmuth, 1990), we argue that once a material element within the cell crosses the *r* = *R*_*p*_ boundary, it translates as a solid body into the pipette without further deformation. Therefore, there are no further gradients in the stresses within the cell projection, such that the stress within the cell at the tip of the cell projection is equal to the stresses at the *r* = *R*_*p*_ boundary.) A slightly different expression applies for Eq. 5b when *L*_*p*_ < *R*_*p*_, as described in supplemental materials, Supplementary Sections S1–S3.

The constitutive equation relates the stresses within the cell to the corresponding rate of deformation. Since we are in principal coordinates, these relationships simplify to (See (Malvern, 1969) (p. 275) or (Fung, 1965) (p. 444):

$$\begin{array}{cc} \tau_{r}=2\mu D_{r} & \tau_{\theta }=2\mu D_{\theta }\tau_{\varphi }=2\mu D_{\varphi } \end{array}$$ (6)

where the (principal) components of the rate of deformation tensor are (Malvern, 1969) (p. 671):

$$\begin{array}{cc} D_{r}=\frac{\partial v_{r}}{\partial r}, & D_{\theta }=D_{\varphi }=\frac{v_{r}}{r} \end{array}$$ (7)

where *v*_*r*_ is the radial material velocity, and incompressibility is assumed. In a departure from previous analyses that treated the cell interior as Newtonian, the viscosity *μ* is taken to be related to the rate of deformation via the power law relationship (Eq. (1)). For the current geometry, the maximum rate of deformation is:

$$D_{\max}=\left|\frac{\partial v_{r}}{\partial r}-\frac{v_{r}}{r}\right|$$ (8)

Conservation of mass and the assumption of incompressibility enables us to relate the radial position and the material velocity *v*_*r*_(*r*) to measurable parameters:

$$\pi C_{Rp}\dot{L_{p}}=-4\pi r^{2}v_{r}$$ (9)

where $\dot{L_{p}}$ is the time derivative of the projection length and *C*_*Rp*_ = *R*_*p*_^*2*^ for *L*_*p*_ ≥ *R*_*p*_ and *C*_*Rp*_ = (*R*_*p*_^2^ + *L*_*p*_^2^)/2 for *L*_*p*_ < *R*_*p*_. Note the sign convention: when the projection length is increasing in size ( $\dot{L_{p}}>0$ ), the radial material velocity is negative (in the direction of the origin). The expression for *μ* is given in Eq. (1), leading to the following expression for the viscous contribution to the change in projection length with time:

$$\frac{dL_{p}}{dt}=\frac{\left(p_{p}-p_{o}\right)+2T_{cort}\left(\frac{1}{R_{p}}-\frac{1}{R_{s}}\right)}{-2\mu_{0}\left(\frac{1}{1-b}\right){\left(\frac{3}{2}\right)}^{-b}{\left(R_{p}\right)}^{2\left(1-b\right)}\left(\frac{1}{{\left(R_{p}\right)}^{3\left(1-b\right)}}-\frac{1}{{\left(R_{s}\right)}^{3\left(1-b\right)}}\right)}$$ (10)

#### 2.3.2 Initial entry diverges from simple theory

**FIGURE 3:**
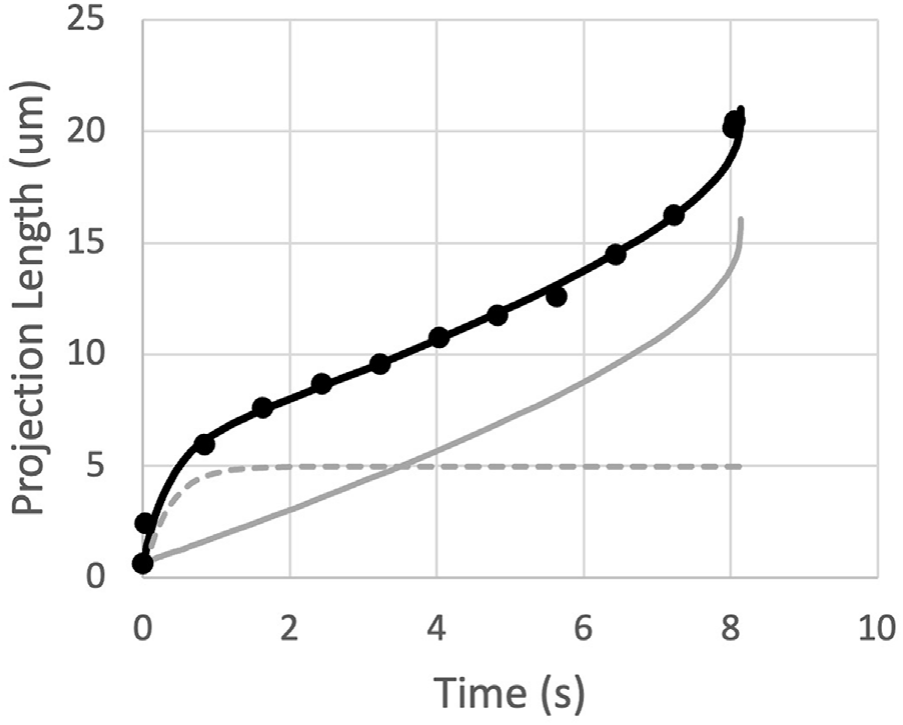
The measured projection length as a function of time is matched by the sum (black curve) of an exponential approach to *L*_*init*_ (dashed gray curve) and the predictions for a power-law fluid (solid gray curve).

Measurements of the time course of entry of naïve T cells into a micropipette reveals an initial rapid entry of the cell into the pipette that is not predicted by the straightforward power-law theory. This behavior is common to all cells and is illustrated in Figure 3. (See also Supplementary Section S4 of the supplemental materials.) The initial entry length (*L*_*entry*_) increases rapidly, but not instantaneously, approaching a limiting value that we designate *L*_*init*_. The time course of this initial entry length can be described empirically as:

$$L_{entry}=L_{init}\left(1-e^{-t/\tau_{init}}\right)$$ (11)

where *τ*_*init*_ is a time constant for cell entry. The time derivative of the initial entry phase is:

$$\frac{dL_{entry}}{dt}=\frac{L_{init}}{\tau_{init}}e^{-t/\tau_{init}}$$ (12)

**FIGURE 4:**
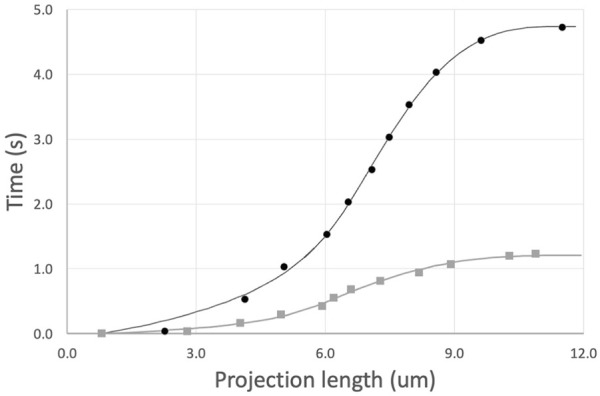
Example fits of two sequential aspirations of a naive T cell. Aspiration pressures were 5 cm H_2_O (top, black curve) and 10 cm (lower gray curve). Power law exponent *b* = 0.50. Fitted parameters and 95% confidence intervals for the top curve, *μ*_*0*_ = 972 ± 51, *τ*_*init*_ = 0.62 ± 0.13, and *L*_*init*_ = 4.96 ± 0.28; and for the bottom curve, *μ*_*0*_ = 876 ± 55, *τ*_*init*_ = 0.12 ± 0.06, and *L*_*init*_ = 4.05 ± 0.39.

The prediction for the complete time course of cell entry is obtained by summing Eqs. 10, 12 and integrating numerically to find *t* (*L*_*p*_). (We use *L*_*p*_ as the independent value to avoid numerical issues during the integration of the time course.) The prediction was matched to individual cell data by least squares regression with three free parameters: *μ*_*0*_, *τ*_*init*_, and *L*_*init*_. Results for two typical fits are shown in Figure 4. This approach provides good agreement with experimental measurements while accounting for position-dependent variations in cell viscosity, when the same cell is aspirated at different pressures to produce different rates of deformation. To determine the shear thinning coefficient *b*, each cell was aspirated at two different pressures, and the value of *b* was adjusted to minimize differences in viscosity across different shear rates, as described in the next section.

#### 2.3.3 Shear thinning data at multiple pressures

The shear thinning behavior was originally discovered by testing cells at different aspiration pressures and observing how both the rate of deformation and the apparent cell viscosity differed when measured at different pressures (Tsai et al., 1993). In the present study, each cell was tested at two different aspiration pressures, providing us the opportunity to observe shear thinning for each cell individually. These data were analyzed by fixing the value of the exponent *b* and then using least squares regression to determine the characteristic viscosity *μ*_*0*_ and the initial entry parameters *τ*_*init*_ and *L*_*init*_. To identify the value of *b* that best characterized the shear dependence of the cell population, we performed the data fits at different values of *b* and selected the one that minimized variation in the values of viscosity across all cells in the population. Measurement variability and differences between different cells resulted in a range of apparent shear thinning behaviors. For each cell, the measured time course of changing projection length leads to an effective viscosity, that in turn can be written in terms of the characteristic viscosity, the power law exponent, and the measured rates of deformation:

$$\mu_{data}=\mu_{o,est}{\left(D_{\max}\right)}^{{-b}_{est}}$$ (13)

(See also Supplementary Section S5 of the supplemental materials.) We can also write this in terms of values for *μ*_*0*_ and *b* that best characterize the behavior of the cell:

$$\mu_{data}=\mu_{o,cell}{\left(D_{\max}\right)}^{{-b}_{cell}}$$ (14)

For a given fixed value of *b*_*est*_, we use least squares regression to obtain two estimations of *μ*_*0,est*_, one at each aspiration pressure. These two values can be used to calculate the difference between the estimated *b* and the value of *b* for this cell:

$$\ln\left(\frac{\mu_{o2,est}}{\mu_{o1,est}}\right)=\left(b_{cell}-b_{est}\right)\ln\left(\frac{D_{\max1}}{D_{\max2}}\right)$$ (15)

We note that the deformation field for the cell is the same regardless of the pressure, meaning that the rate of deformation ***D*** scales in proportion to the inverse of the total time of entry, *t*_*tot*_. Therefore,

$$\ln\left(\frac{\mu_{o2,est}}{\mu_{o1,est}}\right)=\left(b_{cell}-b_{est}\right)\ln\left(\frac{t_{tot2}}{t_{tot1}}\right)$$ (16)

**FIGURE 5:**
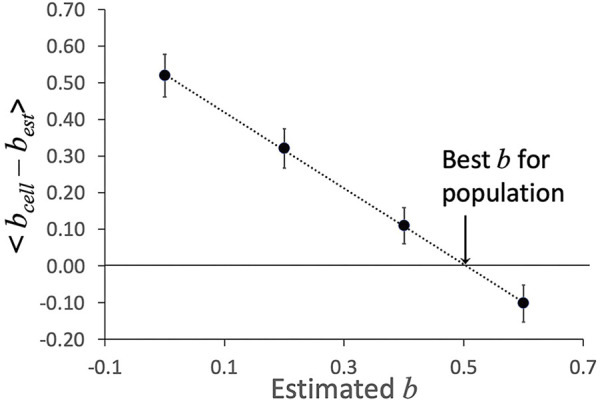
Example of determination of shear thinning coefficient *b*. The average difference < *b*_*cell*_–*b*_*est*_ > was determined at different estimated values of *b*. The value at which the linear fit to these values crosses zero is taken as the “best *b*” for this data set. Error bars show plus or minus standard error of the mean for the 23 cells in this data set.

Procedurally, we set the value for *b*_*est*_ and hold it constant as we analyze all cells in a given experiment. Based on the values of *μ*_*0,est*_ obtained from the fits, and the measured total time of entry at each pressure, Eq. (16) was used to determine *b*_*cell*_ for each cell. The individual cell values were averaged over all cells tested for a given experiment, and an optimal estimate of *b*_*cell*_ was obtained by plotting the average difference < *b*_*cell*_ - *b*_*est*_ > as a function of *b*_*est*_ and taking the value where the line crosses *b*_*cell*_ - *b*_*est*_ = 0 (Figure 5).

Once the “best *b*” value was determined, the data were re-fit with the best value of *b* to obtain best fit values for *μ*_*0*_, *τ*_*init*_, and *L*_*init*_ for each cell in the sample.

### 2.4 Statistical analysis

ANOVA was used to assess significance of differences between different cell groups. For variables that were log-normally distributed, ANOVA was performed on the log of the raw values, and a representative value was defined as exp (<ln(x)>) where < ln(x)> represents the mean of logs of the population values. Significance was assessed at the level of *p* < 0.05.

## 3 Results

Four different T-cell population types were tested: all T cells isolated from peripheral blood (T cells), activated T cells, CD8^+^ cells and activated CD8^+^ cells. Freshly isolated cell samples were tested within 4 hours of phlebotomy, then, whenever possible, cells from the same isolation were activated in culture for seven to 8 days and then tested. For the unfractionated samples, 74 cells from five different isolations were tested prior to activation, and cells from three of these isolations were tested on one or 2 days of experiment approximately 1 week later (total of 5 days and 62 cells). Similarly, freshly prepared CD8^+^ cells were tested on 7 different occasions (total of 133 cells) and cells from five of those isolations were activated in culture and tested seven and 8 days later (total of 10 different days and 97 cells).

### 3.1 Cell size

**FIGURE 6:**
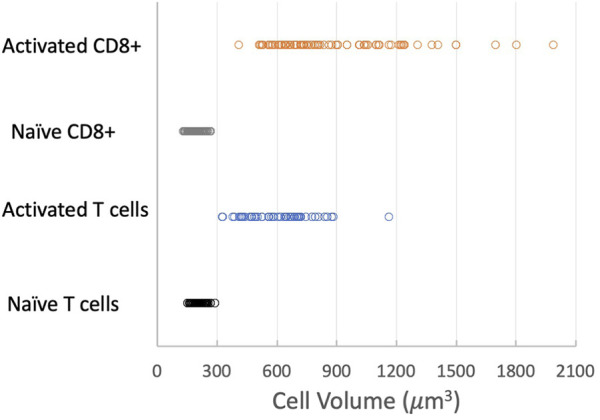
Effect of activation on cell volume. Distribution of cell volumes for naïve T cells (black circles), activated T cells (blue circles), naïve CD8^+^ cells (gray circles) and activated CD8^+^ cells (orange circles). Means, SD, SEM listed in Table 1.

The most significant physical change in the cells upon activation was a change in cell volume. We measured the diameter of each cell prior to being aspirated into the micropipette and calculated the volume from the spherical diameter. The cell volume was normally distributed for a given population of cells (Supplemental materials Supplementary Figure S6). We found that the naïve T-cell population nearly tripled in volume after activation, from a mean of 209 μm^3^ (Standard deviation (SD) = 31.8 μm^3^, Standard Error of the Mean (SEM) = 3.6 μm^3^, n = 74) to a mean of 595 μm^3^ (SD = 156 μm^3^, SEM = 20.0 μm^3^, n = 62). CD8^+^ cells exhibited an even larger increase, more than quadrupling in volume from 194 μm^3^ (SD = 29.8 μm^3^, SEM = 2.6 μm^3^, n = 133) to 858 μm^3^ (SD = 308 μm^3^, SEM = 31.8 μm^3^, n = 95). A comparison of the distribution of values for the four populations tested is shown in Figure 6. Note that the variance in the distribution of cell volumes also increased substantially after activation.

An alternative approach to determining cell volume was to measure the length of the cell once it was fully aspirated inside the pipette (*L*_*tot*_) and calculate the volume of a cylinder (of length *L*_*tot*_ *–2R*_*p*_) with two hemispherical caps: $V=\pi {R_{p}}^{2}L_{tot}-{{2R}_{p}}^{3}/3$ . In our measurements of *L*_*tot*_, we observed that for a given cell measured at two different aspiration pressures, *L*_*tot*_ was generally shorter when the cell was aspirated at the higher pressure, indicating that the volume of the cell decreased with increasing aspiration pressure. We observed that the fractional change in cell volume for a given change in pressure (1/*V dV*/*dP*) varied exponentially with the mean of the two pressures *P =* 0.5(*P*_*1*_ *+P*_*2*_):

$$\frac{1}{V}\frac{dV}{dP}\approx \frac{V_{2}-V_{1}}{P_{2}-P_{1}}\left(\frac{2}{V_{2}+V_{1}}\right)=c_{1}e^{-c_{2}P}\;.$$ (17)

**FIGURE 7:**
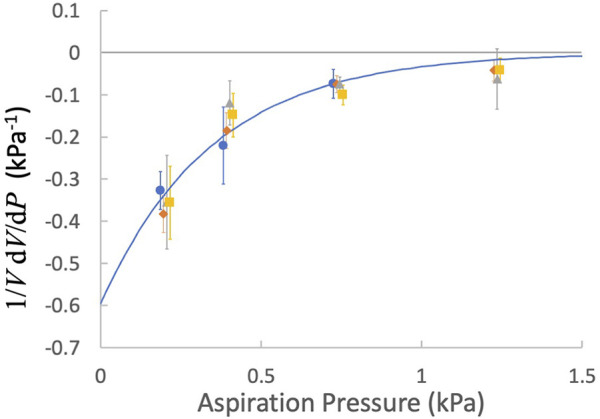
The factional derivative of the cell volume as a function of the aspiration pressure. Points are means and error bars are ± SEM. blue circles, naïve T cells; orange diamonds, naïve CD8^+^ T cells, gray triangles, activated T cells; yellow squares, activated CD8^+^ T cells. Fitted curve: Eq. 17, *c*_*1*_ = −0.60 kPa^-1^ and *c*_*2*_ = 2.88 kPa^-1^.

The fractional volume changes of both naïve and activated cells exhibited similar dependence on pressure. Least squares regression to all data yielded coefficients of *c*_*1*_ = −0.60 ± 0.18 kPa^-1^ and *c*_*2*_ = 2.88 ± 0.84 kPa^-1^, where the ± values indicate 95% confidence bands for the fitted parameters (Figure 7).

Integration of Eq. 17 leads to an expression for the volume as a function of the aspiration pressure:

$$\frac{V\left(P\right)}{V\left(0\right)}=\exp\left[-\frac{c_{1}}{c_{2}}\left(e^{-c_{2}P}-1\right)\right]$$ (18)

where *V* (0) is the cell volume in the absence of an aspiration pressure. We can use Eq. 18 to convert volumes measured at a specific aspiration pressure to an estimate of the cell volume prior to aspiration. These values are tabulated (Table 1) for comparison with the volumes calculated from measurements of *R*_*0*_. Although the means are not identical, the differences between means obtained using the two different methods for the same cell populations were not statistically different. The two populations of naïve cells were also not statistically different from each other, but both activated populations were different from naïve cells and different from each other (ANOVA, *p* < 0.05). (Note that there are twice as many measurements for the calculations based on *L*_*tot*_ because each cell was aspirated into the pipette twice.)

**TABLE 1:** Mean Cell Volumes for Different T-cell Populations.

Volumes (μm^3^) calculated from R0				
	Mean	SD	n	SEM
Naïve T cells	208.7	30.8	74	3.6
Naïve CD8+	194.3	29.8	133	2.6
Activated T cells	595.4	156.1	62	20.0
Activated CD8+	856.5	305.1	97	31.1

Volumes (μm^3^) calculated from *L*_*tot*_ and corrected for aspiration pressure				
	Mean	SD	n	SEM
Naïve T cells	189.4	35.2	148	2.9
Naïve CD8+	152.0	21.3	266	1.3
Activated T cells	549.8	128.1	124	11.5
Activated CD8+	763.6	245.3	194	17.7

### 3.2 Cortical tension

An important property of leukocytes is the cortical tension *T*_*cort*_. This is a contractile force at the cell periphery that tends to minimize the macroscopic area of the cell, and which ultimately accounts for its spherical shape (Evans and Kukan, 1984; Evans and Yeung, 1989). The cortical tension was determined for each cell prior to testing (Eq. 2). The values of *T*_*cort*_ were lognormally distributed (See supplemental materials.) The population of naïve T cells had a representative cortical tension of 142 ± 10 pN/μm (± standard error of the mean, n = 74), and activation did not increase this value significantly (150 ± 14 pN/μm, n = 62). CD8^+^ cells had a similar cortical tension before activation (148 ± 9 pN/μm, n = 133), but *T*_*cort*_ increased significantly after activation (270 ± 18 pN/μm, n = 97). Only activated CD8^+^ cells were statistically different from the other groups (ANOVA, *p* < 0.05). See Table 2 and supplemental materials Supplementary Figure S7.

**TABLE 2:** Summary of material properties of different T cell populations

	T cells Mean ± StdErr	T cells, Activated Mean ± StdErr	CD8+ Mean ± StdErr	CD8+, Activated Mean ± StdErr
Modal Viscosity, *μ*_*0*_ (Pa s)	724 ± 37	1131 ± 72	871 ± 41	1174 ± 62
Initial projection *L*_*init*_/*R*_*p*_	1.51 ± 0.04	2.43 ± 0.08	1.94 ± 0.06	2.84 ± 0.07
Initial Time constant *τ*_*init*_ (s)	0.33 ± 0.04	0.70 ± 0.09	0.42 ± 0.04	0.76 ± 0.08
Power-law coefficient, *b*	0.49 ± 0.03	0.56 ± 0.04	0.54 ± 0.03	0.68 ± 0.03

### 3.3 The initial elastic response

An important deviation from the liquid drop model is the observation by several labs that the initial projection of the cell into the pipette occurs much more rapidly (<0.1 s, typically) than the time course of subsequent viscous entry. In this analysis we capture this in terms of an exponential approach to an initial length *L*_*init*_. The values of *L*_*init*_ were normally distributed. (See supplemental material, Supplementary Figure S8). The mean *L*_*init*_ for naive T cells (3.41 ± 0.08 μm) was not statistically different from CD8^+^ T cells (3.89 ± 0.09 μm). Activation, however, led to a significant increase in *L*_*init*_. For activated T cells, *L*_*init*_ increased more than twofold to 7.41 ± 0.23 μm, and for the CD8^+^ population, *L*_*init*_ increased approximately 2.5-fold–9.68 ± 0.25 μm. The distribution of values was also broader for the activated populations, reflecting increased heterogeneity.

### 3.4 Dependence of *L*_*init*_ on pressure

It is generally thought that the initial rapid entry reflects an elastic response of the cell to sudden imposition of the aspiration pressure. For a simple elastic material, it is expected that application of larger forces ought to result in larger deflections, all else being equal. Surprisingly, we do not find that the initial projection length depends on the applied pressure to a significant extent. To avoid artifactual effects of pipette size, we examined whether the same cell being aspirated into the same pipette had different initial projection lengths at the two different aspiration pressures. Analogous to our approach to reveal the dependence of cell volume on aspiration pressure, for each cell we calculated:

$$\frac{dL_{init}}{dP}\approx \frac{L_{init,1}-L_{init,2}}{P_{1}-P_{2}}$$ (19)

The distribution of these values was normal for all 4 cell populations tested, and in each case the mean value was not statistically different from zero (supplemental material, Supplementary Figure S8). Neither was there a significant dependence of the derivative on pressure, in contrast to the results obtained for the cell volume. Therefore, contrary to expectation, we could not detect a significant dependence of the initial projection length on pressure.

To ensure that this was not the effect of compensating factors, like cell or pipette size, we estimated a dimensionless extension ratio at the tip of the micropipette based on the deformation of a series of disc-shaped slices of a sphere moving into a pipette at constant volume. (See supplemental materials, Supplementary Figure S6.) We designate this quantity as *λ*_*tip,init*_, and examined d*λ*_*tip,init*_/d*P* as a distribution and as a function of pressure. The behavior was the same as we observed for *L*_*init*_. The distribution was normal with a mean indistinguishable from zero, and there was no dependence of the quantity on pressure (not shown).

### 3.5 Viscosity and shear thinning

As captured in Eq. (1), there are two parameters that characterize the cells resistance to flow, the characteristic viscosity *μ*_*o*_ and the shear thinning coefficient *b*. The characteristic viscosity was lognormally distributed (see supplemental material, Supplementary Figure S9), and statistical significance was assessed using the log of the coefficients. The representative viscosity was higher for CD8^+^ cells (870 ± 41 Pa s, n = 266) than naïve T cells (722 ± 37 Pa s, n = 148), and activation resulted in increased viscosity for both cell types (activated CD8^+^, 1,174 ± 62 Pa s, n = 194; activated T cells, 1,186 ± 85 Pa s, n = 118). One-way ANOVA analysis revealed that the viscosity of naive T cells was significantly different from that of naïve CD8^+^ cells, and that naïve cells have significantly lower viscosity than either of the activated cell groups, which were not significantly different from each other (*p* < 0.05). The shear thinning coefficient *b* calculated for individual cells was normally distributed (supplemental materials Supplementary Figure S10). The mean value *b* for T cells was 0.50 ± 0.03, n = 74, and this was not significantly different from the mean for naïve CD8^+^ cells, 0.54 ± 0.03, n = 133. Activation of T cells resulted in a small, but not statistically significant, increase in *b,* 0.55 ± 0.04, n = 62, but the increase after activation of CD8^+^ cells was significant, 0.66 ± 0.03, n = 97.

## 4 Discussion

### 4.1 Importance of T cell mechanical properties, relevance to cell therapeutics

It has been known for some time that activated leukocytes can block capillaries, compromising the microcirculation and tissue oxygenation (Worthen et al., 1989). Using a novel single cell rheometer, Zak et al., 2021 have demonstrated rapid increases in resistance to deformation upon activation in T cells and B cells. Changes in cell properties have also been documented in different clinical conditions, including sepsis (Nishino et al., 2005), diabetes (Perrault et al., 2004), or radiation exposure (Thomas et al., 2003). Understanding of the changes in leukocytes (T cells in particular) with activation has taken on particular clinical relevance with the growth of cell therapeutics.

During *in vitro* activation and proliferation, T cells dramatically increase in size. This process is often associated with significant changes in the membrane topography and increased microvilli density with a dynamic reorganization of the intracellular cytoskeletons. In this study, we demonstrate the reduced deformability of activated T cells, as measured by elevated cortical tension and dynamic resistance to deformation (viscosity). Therefore, it is likely that these combined effects of therapeutically manufactured T cells would lead to substantially increased resistance to passage through the microvasculature after infusion, causing the inefficient trafficking of the transferred T cells to the target tissue site.

We note that naïve lymphocytes, although smaller than neutrophils, have higher resistance to deformation, both in terms of cortical tension, which is ten times larger than neutrophils (Needham and Hochmuth, 1992), and in characteristic viscosity, which is roughly four times larger (Evans and Yeung, 1989). Properties of T cells more closely resemble those of a monocyte cell line (J774) (Lam et al., 2009) or hybridoma cells (Needham et al., 1991). This suggests that neutrophils tend to be softer than most suspension cells, consistent with the role of the neutrophil as a highly motile cell capable of facile movement into and through extravascular spaces.

### 4.2 Cell volume changes

A novel finding of the present study is the observation that the cell volume decreases monotonically with increasing aspiration pressure. In red cells, such a volume decrease was identified and explained in terms of non-equilibrium thermodynamics (Evans and Waugh, 1977). The application of pipette suction creates a pressure gradient between the cell interior and the surrounding fluid, causing water to flow out of the cell. As water leaves, the concentration of ions inside the cell increases, creating an osmotic gradient that tends to pull water back into the cell. At steady state, water flows through the cell: into the cell from the suspending medium outside the pipette and out of the cell across the membrane inside the pipette. When we considered a similar model to explain the changes in leukocyte volume, we found that the predicted change in volume was linear with increasing pressure, inconsistent with the exponential behavior we observe. (See supplemental materials, Supplementary Figure S7) We conclude that a simple osmotic model does not provide an adequate explanation for this effect. Rather, we hypothesize that the tendency for water to leave the cell is opposed by an elastic resistance of the cytoskeleton to compression. Evaluation of this hypothesis awaits further study.

There is evidence in the literature that decreasing the volume of an individual cell can lead to increased stiffness, as assessed by magnetic twisting cytometry (Guo et al., 2017). Our observation that T cell volume decreases at higher aspiration pressures suggests that there could be a corresponding increase in cell stiffness. This might lead to an underestimation of shear thinning as at higher shear rates (higher aspiration pressures) the loss in volume could lead to an elevation of cell viscosity above what the same cell might exhibit with a slightly larger volume. It is important to note that our result that activated cells (with larger volumes) have larger cortical tension and larger viscosities than their smaller naïve counterparts does not contradict prior observations that reducing the volume of a particular cell increases its stiffness. Our results show that for different types of cells (naïve vs. activated) there is not a general correlation that larger cells are necessarily “softer.” Indeed, our results are in agreement with other prior studies that showed HL60 cells in the S phase of the cell cycle are larger and stiffer than their G1 counterparts (Tsai et al., 1996). Thus, while for an individual cell, stiffness is expected to increase with decreasing cell volume, this expectation does not hold when comparing cells of different type or different state.

### 4.3 Modeling descriptions of leukocytes: (visco)elastic solids vs. viscous drops

Competing descriptions of leukocyte rheology date back 4 decades. In one of the earliest mechanical models, the cell was described as a viscoelastic solid (Schmid-Schonbein et al., 1981). This description of the cell appeared to work well for small deformations of relatively short duration, but it has been largely superseded by a model of the cell as a viscous droplet with a contractive “tension” at the cell cortex. In this model, the cortical tension is responsible for restoring cells to a spherical shape after deformation, and the cell interior is treated as a simple viscous fluid (Evans and Kukan, 1984; Evans and Yeung, 1989; Needham and Hochmuth, 1990; Tran-Son-Tay et al., 1991; Needham and Hochmuth, 1992). This model better accounts for the macroscopic behavior of the cell for large deformations and long times, although it fails to account for the initial rapid entry phase during leukocyte aspiration into a micropipette. An extension of this model to account for shear thinning behavior was subsequently introduced (Tsai et al., 1993; Drury and Dembo, 2001). Another important description of the cell is that of a Maxwell fluid (Dong et al., 1988). This model accounts for the initial elastic extension and approximates longer term flow behavior, but the analysis is valid only for small deformations.

The development of these models of leukocyte behavior were based on experiments performed on primary human neutrophils, although several other cell types have been found to exhibit similar behavior (Needham et al., 1991; Tsai et al., 1996; Lam et al., 2009). Studies of the mechanical properties of lymphocytes are far less common. T cell viscosity and cortical tension have been reported on the range of 500 Pas, increasing to ∼1,000 Pas after experiencing radiation insult (Thomas et al., 2003), in good agreement with the numbers we report here. More recent reports on T cell mechanical properties treated the cell as an elastic solid (Bufi et al., 2015; Esteban-Manzanares et al., 2017), a model that does not capture cell behavior except for small deformations and short times. In one recent report, investigators used atomic force microscopy to perform small indentations of T cells and uses Hertz theory and a Maxwell fluid model as a descriptor for cell behavior (Li et al., 2016). After an initial elastic response, an additional relaxation was observed, and the cell response was characterized in terms of an elastic modulus plus two relaxation time constants. This approach is more appealing than the simple elastic theories because it accounts for continued cell deformation under constant force. However, the mismatch in geometry between the Hertz model (which assumes a semi-infinite elastic body) and the cell (which is a sphere) limits this approach to very small cell deflections. Moreover, all these models neglect contributions from the cortical tension of the cell, which for small deflections could make a substantial contribution to the cellular response. Therefore, results from these more recent studies cannot be compared directly to the results presented here except in the most general terms.

### 4.4 The initial response

One important aspect of cellular behavior that is not accounted for by the liquid droplet model is the rapid initial phase of cell entry. We and others (Schmid-Schonbein et al., 1981; Herant et al., 2003) attribute this to some kind of elastic behavior of the cell over a short timeframe, but an elastic behavior has not been formally integrated into a model that accounts for cell behavior at longer times and larger deformations, to our knowledge. This shortcoming takes on greater significance in light of our findings that the length of the initial projection increases significantly after cell activation. Understanding the physical basis for this rapid entry phase and developing a unified model that includes this elastic behavior in a way that is not contradicted by cell behavior at larger deformations is an important need for the field. If the initial entry into the pipette is an elastic response, it appears that must involve rapid stiffening because we could not find conclusive evidence that the magnitude of the response increases with increasing pressure. This is but one of the challenges that will need to be overcome to develop a more comprehensive description of cell mechanical behavior.

### 4.5 Cell cortex as a contributor to resistance to deformation

Several investigators have posed the possibility that the primary resistance to leukocyte deformation lies in the cell cortex and is not distributed uniformly throughout the cell interior (Dong et al., 1988; Yeung and Evans, 1989; Herant et al., 2003). In particular, Herant et al. (2003) proposed an unfolding of the cell cortex as a possible explanation for the initial rapid phase of cell entry. Based on our values determined for *L*_*init*_, we calculate that the initial fractional change in area for T cells would be approximately 7% and slightly larger, approximately 12%, for CD8^+^ cells, and larger for activated cells: about 25% for activated T cells and approaching 40% for activated CD8^+^ cells. These experimental estimates are much larger than originally posited by (Herant et al., 2003), but still fall within the maximum area expansions (∼100%) measured during osmotic swelling (Ting-Beall et al., 1993), suggesting that whatever unfolding might occur is easily within the capacity of the cell to increase its area, and therefore, is unlikely to account for the abrupt change in cell behavior after the initial entry. The most rigorous test of whether resistance of the cell cortex governs cell behavior was done by (Yeung and Evans, 1989), who used measurements of cell entry into different-sized pipettes and an accompanying analysis of core and cortical flow of an aspirated cell to demonstrate that dissipation of the cell interior dominates the entry process (Yeung and Evans, 1989). Based on that study, we believe it is unlikely that resistance to area changes (and accompanying resistance to deformation of the cell cortex) is responsible for the large initial entry of the cell.

### 4.6 Concluding remarks

The diversity of models used to describe the response of cells to mechanical forces makes it difficult to make quantitative comparisons among publications using different theoretical frameworks. We have chosen to use the liquid droplet model with shear thinning because of its wide acceptance compared with other models, and the fact that it provides a satisfactory description of cell behavior, with the notable exception of the initial quasi-elastic response of cells to a sudden imposition of force. What is clear from our studies is that activation leads to a significant increase in the size and dynamic stiffness (viscosity) of T cells. Most interesting is the surprising result that activated cells appear to exhibit greater elastic compliance in response to the sudden imposition of force, a characteristic that warrants further investigation in terms of mechanical modelling. The result is also interesting from a physiological perspective as the increased compliance may reflect a greater propensity to exhibit spontaneous shape changes such as those associated with cell motility.

## Data Availability

The data are archived at the Open Science Forum website at the following link: https://osf.io/xqbca/.
