# Supplementary material for: Activation effects on the physical characteristics of T lymphocytes
Source: Front Bioeng Biotechnol. 2023 May 15;11:1175570. doi: 10.3389/fbioe.2023.1175570 (PMC10225623; doi:10.3389/fbioe.2023.1175570)
Supplement: Supplementary file 1 [file DataSheet1.PDF]

Activation Effects on the Physical Characteristics of T Lymphocytes

Richard E. Waugh [1*], Elena Lomakina [1], Andrea Amitrano [2], Minsoo Kim[2]

[1] Department of Biomedical Engineering, University of Rochester, Rochester, NY, USA
[2] Department of Microbiology and Immunology, University of Rochester, Rochester, NY, USA

Correspondence:
Richard E. Waugh
richard.waugh@rochester.edu

List of symbols used in the manuscript

Symbol	Units	Description
b	dimensionless	Power-law shear-thinning coefficient
b_{cell}	dimensionless	True value of *b* for a cell
b_{est}	dimensionless	Trial value of *b* used in least squares regression
c_1	Pa^{-1}	Constant used to describe the dependence on cell volume on aspiration pressure
c_2	Pa^{-1}	Constant used to describe the dependence on cell volume on aspiration pressure
C_{Rp}	μm^2	Coefficient used to relate time projection length velocity to material velocity
D_{max}	s^{-1}	Maximum shear rate
D_r	s^{-1}	Principal shear rate in radial direction
D_θ	s^{-1}	Principal shear rate in azimuthal direction
D_φ	s^{-1}	Principal shear rate in meridional direction
L_{entry}	μm	Initial entry length of the cell projection
L_{init}	μm	Asymptotic value for initial entry length
L_p	μm	Length of projection in the micropipette
$\dot{L}_p$	μm/s	Rate of change of the projection length in the micropipette
L_{tot}	μm	Total length of a fully aspirated cell inside the pipette
P	Pa (pN/μm^2)	Mean aspiration pressure for a cell
P_1	Pa (pN/μm^2)	First pressure at which a cell is aspirated
P_2	Pa (pN/μm^2)	Second pressure at which a cell is aspirated
p_o	Pa (pN/μm^2)	Pressure in the suspending fluid outside the cell
p_p	Pa (pN/μm^2)	Pressure in the lumen of the micropipette
r	μm	Radial coordinate
R_h	μm	Instantaneous hemispherical radius of the cell outside the pipette
R_o	μm	Radius of the cell prior to being aspirated into the micropipette
R_s	μm	Instantaneous spherical radius of the cell outside the pipette
t	s	Time
T_{cort}	pN/μm	Cortical tension of the cell
t_{tot}	s	Total time of cell entry
V	μm^3	Cell Volume
V_1	μm^3	Cell volume after its first aspiration (based on L_{tot})

V_2	μm^3	Cell volume after its second aspiration (based on L_{tot})
v_r	μm/s	Material velocity on the radial direction
$\lambda_{tip,init}$	dimensionless	Estimated material extension ratio at the tip of the micropipette for projection length L_{init}.
μ	Pa s	Cell viscosity
μ_{data}	Pa s	Cell viscosity based on the time course of the aspiration
μ_o	Pa s	Characteristic cell viscosity
$\mu_{o,cell}$	Pa s	True characteristic viscosity of the cell
$\mu_{o,est}$	Pa s	Estimated viscosity of the cell based on b_{est}
σ_{rr}	Pa (pN/μm^2)	Total stress in the radial direction
$\boldsymbol{\tau}$	Pa (pN/μm^2)	Deviatoric material stress tensor
τ_r	Pa (pN/μm^2)	Principal deviatoric material stress in the radial direction
τ_φ	Pa (pN/μm^2)	Principal deviatoric material stress in the φ-direction
τ_θ	Pa (pN/μm^2)	Principal deviatoric material stress in the θ-direction
τ_{init}	s	Time constant for an exponential approach to L_{init}.

Additional symbols used in the supplemental materials

Symbol	Units	Description
A	μm^2	Area
A_o	μm^2	Area of cell outside the pipette
A_p	μm^2	Area of the cell inside the pipette available for water transport
c_o	moles/μm^3	Concentration of solute in the suspending medium
$C_{\lambda r}$	μm^3	Coefficient used in calculating material extension ratios
k	μm^3 s^{-1} Pa^{-1}	Filtration coefficient (Includes area over which flux occurs)
L_{pmin}	μm	Axial length of the overlap of the spherical cell with the pipette opening
$\dot{m}_w$	μm^3 s^{-1}	Volume flux of water across the cell membrane
n_s	moles	Number of solute molecules inside the cell
p_c	Pa	Pressure inside the cell (neglecting elastic contributions)
r_o	μm	Radial coordinate in the undeformed state
R_{cp}	μm	Radius of curvature of the cell projection inside the pipette
RT	J/mole	Gas constant times temperature
R_w	dimensionless	Fraction of the cell volume that is osmotically active
V_{bo}	μm^3	Volume of the intersection of the spherical cell with the pipette opening
V_p	μm^3	Volume of the cell portion inside the pipette
z	μm	Axial position of a material element in cylindrical coordinates
z_o	μm	Axial position of the undeformed material element
Δp	Pa	Pressure difference between suspending medium and pipette lumen
λ_r	dimensionless	Material extension ratio in the radial direction
λ_φ	dimensionless	Material extension ratio in the azimuthal direction
λ_θ	dimensionless	Material extension ratio in the meridional direction
χ_{sc}	dimensionless	Mole fraction of solute inside the cell
χ_{so}	dimensionless	Mole fraction of solute outside the cell
χ_{sc0}	dimensionless	Mole fraction of solute inside the cell in the resting state

Section S1. Kinematics of cell entry

The relationships between the length of the cell projection into the pipette and the intracellular material velocities and extensions is fundamental to making experimental predictions based on material models. When a spherical cell meets the cylindrical opening of the micropipette the sphere extends a short distance into the pipette even before any deformation takes place (Figure S1.) This distance, L_{pmin}, is calculated by simple application of the Pythagorean theorem:

$$L_{pmin} = R_o - \sqrt{{R_o}^2 - {R_p}^2} \qquad (S1)$$

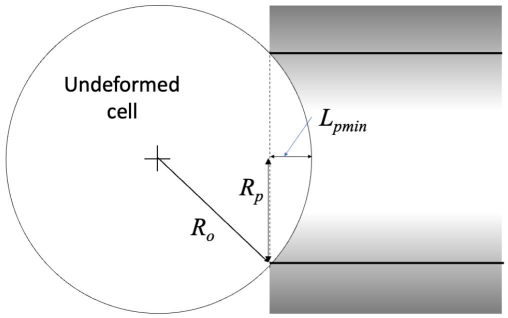

Figure S1. Schematic showing the initial projection of spherical cell into the micropipette prior to cell deformation.

The corresponding volume of this spherical section is given by:

$$V_{bo} = \frac{\pi L_{pmin}}{6}\left(3{R_p}^2 + {L_{pmin}}^2\right) \qquad (S2)$$

As the cell begins to deform, the rate of change of the volume of the cell inside the pipette increases as

$$\frac{dV_p}{dt} = \pi C_{Rp}\dot{L_p} \qquad (S3)$$

where

$$C_{Rp} = \left({R_p}^2 + {L_p}^2\right)/2 \qquad L_p < R_p$$
$$C_{Rp} = {R_p}^2 \qquad L_p \geq R_p$$

From conservation of mass, and assuming that the volume of the cell is incompressible, this change in volume must be matched by the flux across any spherical boundary (or in the case of the hemispherical model, hemispherical boundary) inside the cell. For the sphere,

$$\frac{dV_p}{dt} = -4\pi r^2 v_r \qquad R_p \leq r \leq R_s(t) \qquad (S4)$$

For the hemisphere, 4π is replaced by 2π, and R_s is replaced with R_h, the radius of the hemisphere. Combining equations 3 and 4 we obtain

$$v_r = -\frac{C_{Rp}\dot{L_p}}{4r^2} \qquad (for\ a\ sphere) \qquad (S5a)$$

$$v_r = -\frac{C_{Rp}\dot{L_p}}{2r^2} \qquad (for\ a\ hemisphere) \qquad (S5b)$$

To calculate the magnitude of the extension, one must keep track of the original position of a material element. For a material element originally at radial distance r_o from the center of the sphere that is a distance r from the center of the sphere in the deformed cell, the azimuthal and polar material extension ratios λ_φ and λ_θ are

$$\lambda_\varphi = \lambda_\theta = \frac{r}{r_o} \qquad (S6a)$$

and for an incompressible material,

$$\lambda_r = \frac{1}{\lambda_\varphi \lambda_\theta} = \left(\frac{r_o}{r}\right)^2 \qquad (S6b)$$

To relate the instantaneous position of an element r to its original position r_o, we simply need to keep track of the volume displaced into micropipette from the center of the sphere. Recall from Figure S1 and Equations S1 and S2, there is a small portion of the sphere inside the pipette before the cell begins to deform. Therefore, the displaced volume between r_o and r is equal to the volume in the micropipette minus the initial volume:

$$\frac{4\pi}{3}\left(r_o{}^3 - r^3\right) = \frac{\pi L_p}{6}\left(3R_p{}^2 + L_p{}^2\right) - V_{bo} \qquad L_{pmin} \le L_p < R_p \qquad (S7a)$$

$$\frac{4\pi}{3}\left(r_o{}^3 - r^3\right) = \pi R_p{}^2\left(L_p - R_p/3\right) - V_{bo} \qquad L_p \ge R_p \qquad (S7b)$$

Algebraic manipulation leads to:

$$\lambda_r = \left(1 + \frac{C_{\lambda r}}{r^3}\right)^{2/3} \qquad (S8)$$

where

$$C_{\lambda r} = \frac{L_p}{8}\left(3R_p{}^2 + L_p{}^2\right) - \frac{3V_{bo}}{4\pi} \qquad L_{pmin} \le L_p < R_p$$

$$C_{\lambda r} = \frac{R_p{}^2}{4}\left(3L_p - R_p\right) - \frac{3V_{bo}}{4\pi} \qquad L_p \ge R_p$$

We will use these relationships in the following sections, with appropriate adjustments for the hemisphere or collapsing sphere models.

Section S2: Predictions for the power-law fluid model using hemispherical approximation of the cell outside the pipette.

As originally proposed by Needham and Hochmuth [1], this analysis replaces the spherical outer portion of the cell with a hemisphere of the same volume. The geometrical approximation vastly simplifies analysis of the deformation field inside the cell as it enters the micropipette. The original treatment by Needham and Hochmuth assumed a Newtonian fluid. Here we introduce the shear-thinning power-law fluid model, which accounts for variations in the local viscosity based on the location-dependent shear rate.

Setting the origin of a spherical coordinate system at the center of the mouth of the pipette (Figure S2), we observe that the deformation is invariant for both angular coordinates, θ and φ. We consider the flow field between the outer boundary of the cell, $r = R_h$, and a hemispherical boundary of radius R_p (the pipette radius) at the tip of the pipette. Recognizing the symmetry of the problem, and neglecting inertial and gravitational terms, the equation of motion, $\nabla p = -\nabla \cdot \boldsymbol{\tau}$, becomes [2]

$$\frac{\partial p}{\partial r} = \frac{1}{r^2}\frac{\partial}{\partial r}\left(r^2 \tau_{rr}\right) - \frac{\tau_{\theta\theta} + \tau_{\varphi\varphi}}{r} \qquad (S9)$$

where $\boldsymbol{\tau}$ is the shear stress tensor and p is the pressure. (Note: the sign convention for pressure is opposite that used in the reference [2] In keeping with the concept of the cell as a fluid droplet with a cortical tension T_{cort}, we recognize that a force balance can be written at the outer boundary ($r = R_s(t)$):

$$-\sigma_{rr}|_{r=R_s} = p_o + \frac{2T_{cort}}{R_s} \qquad (S10a)$$

and at the inner boundary:

$$-\sigma_{rr}|_{r=R_p} = p_p + \frac{2T_{cort}}{R_{cp}} \qquad \text{(S10b)}$$

where R_{cp} is the radius of curvature of the cell boundary inside the pipette, and the total stress within the cell is $\sigma_{rr} = -p + \tau_{rr}$. We assume that once a material element within the cell crosses the $r = R_p$ boundary, it translates as a solid body into the pipette without further deformation. Therefore, there are no further gradients in the stresses within the cell projection, such that the stress within the cell at the tip of the cell projection in the pipette are equal to the stresses at the $r = R_p$ boundary. This is not strictly the case when $L_p < R_p$, and the hemispherical shape of the tip of the projection inside the pipette is still being formed, but we neglect this small additional deformation in our analysis. We do, however, account for the change incurvature of the cell interface inside the pipette when $L_p < R_p$:

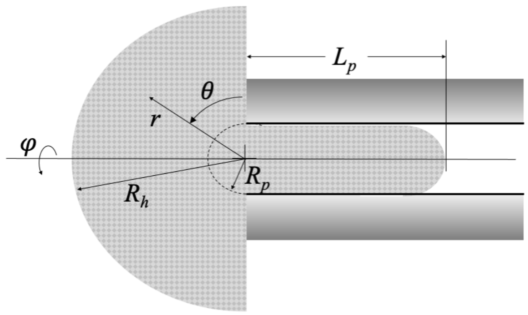

Figure S2. Schematic of the "mushroom" approximation of a cell entering a micropipette. The radius of the pipette is R_p, the radius of the hemisphere is R_h, and the length of the projection in the pipette is L_p. Spherical coordinates r, θ, φ are centered at the center of the pipette opening. Deformation occurs in the region between the outer boundary, $r = R_h$ and an inner boundary at $r = R_p$.

$$R_{cp} = R_p \ , \qquad L_p \geq R_p \qquad (S11a)$$

$$R_{cp} = \left({R_p}^2 + {L_p}^2\right)/2L_p \ , \qquad L_p < R_p \qquad (S11b)$$

The constitutive equation relates the stresses within the cell to the corresponding rate of deformation. The (principal) components of the rate of deformation tensor ***D*** are [3]:

$$D_r = \frac{\partial v_r}{\partial r}, \qquad D_\theta = D_\varphi = \frac{v_r}{r} \ . \qquad (S12)$$

The maximum rate of deformation is:

$$D_{max} = \left|\frac{\partial v_r}{\partial r} - \frac{v_r}{r}\right| . \qquad (S13)$$

Using a power law relationship for a shear-rate dependent viscosity:

$$\mu = \mu_o (D_{max})^{-b} \ , \qquad (S14)$$

we obtain the constitutive relationships:

$$\tau_r = 2\mu_o \left(\frac{\partial v_r}{\partial r} - \frac{v_r}{r}\right)^{-b} \frac{\partial v_r}{\partial r} \qquad (S15)$$

$$\tau_\theta = \tau_\varphi = 2\mu_o \left(\frac{\partial v_r}{\partial r} - \frac{v_r}{r}\right)^{-b} \frac{v_r}{r} \qquad (S16)$$

where μ_o is the characteristic viscosity (when D_{max} = 1) and b is the power-law fluid exponent. Note that the second subscript has been dropped because these are principal components of the tensors $\boldsymbol{\tau}$ and $\boldsymbol{D}$. Our assumption that the cell is incompressible is used to establish a relationship between the material velocity v_r and radial position r (Equation S5b). Note the sign convention: when the projection length is increasing in size ($\dot{L_p} > 0$), the radial material velocity is negative (in the direction of the origin). Substituting this relationship for v_r and evaluating, we obtain the following expressions for the principal stress components:

$$\tau_r = 2\mu_o \left(\frac{3C_{Rp}\dot{L_p}}{2r^3}\right)^{-b} \frac{C_{Rp}\dot{L_p}}{r^3} \qquad (S17)$$

and

$$\tau_\theta = \tau_\varphi = -2\mu_o \left(\frac{3C_{Rp}\dot{L_p}}{2r^3}\right)^{-b} \frac{C_{Rp}\dot{L_p}}{2r^3}. \qquad (S18)$$

Substituting these expressions into the equation of motion, we obtain:

$$\frac{\partial p}{\partial r} = 6\mu_o b \left(\frac{3C_{Rp}\dot{L_p}}{2r^3}\right)^{-b} \frac{C_{Rp}\dot{L_p}}{r^4} \;. \qquad (S19)$$

To obtain the solution, we integrate the above expression subject to the boundary conditions (Eq. S10). Integrating Eq. S19, we obtain:

$$\left(\frac{dL_p}{dt}\right)^{(1-b)} = \frac{(p_o - p_p) - 2T_{cort}\left(\frac{1}{R_{cp}} - \frac{1}{R_h}\right)}{2\mu_0\left(\frac{1}{1-b}\right)\left(\frac{3}{2}\right)^{-b}\left(C_{Rp}\right)^{(1-b)}\left(\frac{1}{\left(R_p\right)^{3(1-b)}} - \frac{1}{(R_h)^{3(1-b)}}\right)} \qquad (S20)$$

Where R_{cp} is the radius of curvature of the cell projection edge inside the pipette:
This equation can be used to generate predictions for L_p as a function of time. Note that increasing b increases the acceleration of the cell near the end of its entry into the pipette. Setting b = 0 recovers the solution originally published by Needham and Hochmuth [1].

Section S3. Power law fluid model for a collapsing sphere.
The derivation for the collapsing sphere approximation is very similar to the hemispherical model, except that the outer boundary is a sphere of radius R_s, and the expression for the material velocity (Eq. S7) differs by a factor to two:

$$\pi C_{Rp}\dot{L_p} = -4\pi r^2 v_r \qquad (S21a)$$

or

$$v_r = -\frac{C_{Rp}\dot{L_p}}{4r^2} \;, \qquad (S21b)$$

Carrying through the calculations, we ultimately arrive at the following expression for the time derivative of L_p:

$$\left(\frac{dL_p}{dt}\right)^{(1-b)} = \frac{(p_o - p_p) - 2T_{cort}\left(\frac{1}{R_{cp}} - \frac{1}{R_h}\right)}{\left(\frac{\mu_0}{1-b}\right)\left(\frac{3}{4}\right)^{-b}\left(C_{Rp}\right)^{(1-b)}\left(\frac{1}{\left(R_p\right)^{3(1-b)}} - \frac{1}{(R_h)^{3(1-b)}}\right)} \qquad (S22)$$

Section S4. Simple Power-law model fails to capture initial rapid entry: ad hoc correction.
Measurements of the time course of entry of naïve T cells and passive neutrophils into a micropipette reveals an important discrepancy between the straightforward power-law theory developed in previous publications (and detailed in sections 1 and 2) and actual cell behavior. This is illustrated in Figure 3 of the manuscript. The simple viscous theory prediction is shown as the solid gray curve, which starts almost linearly from the initial value of the projection length and fails to capture the rapid initial entry of the cell into the pipette. Importantly, all cells exhibited a similar initial rapid

entry that is not predicted by the simple theory. This initial entry length L_{entry}, increases rapidly, but not instantaneously, with a time course that appears to be exponential, approach a limiting value that we designate L_{init}. The time course of this initial entry length can be described empirically as:

$$L_{entry} = L_{init}\left(1 - e^{-t/\tau_c}\right) \qquad (S23)$$

where τ_c is a time constant. The time derivative of the initial entry phase is:

$$\frac{dL_{entry}}{dt} = \frac{L_{init}}{\tau_c} e^{-t/\tau_c} \qquad (S24)$$

The prediction for the complete time course of cell entry is obtained by summing Eqs. S22 and 24 and integrating numerically to find $t(L_p)$. The prediction was matched to individual cell data by least squares regression with three free parameters: μ_0, L_{init}, and τ_c.

Section S5. Determination of power law coefficient for individual cells.

To best understand the dependence of the cell viscosity on shear rate, the approach was taken to measure each cell at two different aspiration pressures, that resulted in two different rates of deformation. The geometry of cell entry is the same for repeated measurements on the same cell, whereas it may differ for different cells because of small differences in cell size. Properties may also differ from cell to cell. By testing each cell at two different aspiration pressures, the influence of cell-to-cell variability on the determination of b was minimized so that the dependence of viscosity on shear rate is most clearly revealed. In the analysis, the power-law coefficient is fixed at a value b_{est}, and a corresponding value for the characteristic viscosity was determined for each aspiration data set. If b_{est} is equal to the true shear thinning coefficient b_{cell} then the calculated values of the characteristic viscosity at the two different aspiration pressures μ_{o1} and μ_{o2} should be equal. If they are not equal, then the difference between b_{est} and b_{cell} can be estimated as follows.

Taking the characteristic shear rate to be 1.0, the measured viscosity for aspiration pressure 1 ($\mu_{meas,1}$) is related to the true characteristic viscosity of the cells $\mu_{o,cell}$ as:

$$\mu_{meas,1} = \mu_{o,cell}\left(D_{max,1}\right)^{-b_{cell}} \qquad (S25)$$

and for the second aspiration pressure:

$$\mu_{meas,2} = \mu_{o,cell}\left(D_{max,2}\right)^{-b_{cell}} \qquad (S26)$$

Similar relationships exists between the viscosities based on measurement and the estimated value b_{est} and the corresponding estimations of the characteristic viscosity $\mu_{o1,est}$ and $\mu_{o2,est}$:

$$\mu_{meas,1} = \mu_{o1,est}\left(D_{max,1}\right)^{-b_{est}} \qquad (S27)$$

$$\mu_{meas,2} = \mu_{o2,est}\left(D_{max,2}\right)^{-b_{est}} \qquad (S28)$$

The values for $\mu_{meas,1}$ and $\mu_{meas,2}$ are based on experimental measurement and must be the same in each of the two expressions in which they appear. Furthermore, the true characteristic viscosity of the cell $\mu_{o,cell}$ is also invariant. Using these arguments, we can manipulate Eqs S25-S28 to obtain:

$$\frac{\mu_{o2,est}}{\mu_{o1,est}} = \left(\frac{D_{max,1}}{D_{max,2}}\right)^{(b_{cell}-b_{est})} \qquad (S29)$$

These relationships are exact for homogeneous deformations and are approximate for cell aspiration experiments where the maximum shear rate D_{max} varies with position and time. We argue that because the dimensions of the cell and the micropipette are the same for both aspirations, deformation of the cell is the same for both aspiration pressures, and the maximum shear rate should scale as the inverse of the total time for cell entry t_{tot}:

$$\frac{\mu_{o2,est}}{\mu_{o1,est}} = \left(\frac{t_{tot,2}}{t_{tot,1}}\right)^{(b_{cell}-b_{est})} \qquad \text{(S30)}$$

Taking the natural log and re-arranging, we obtain Equation 15 of the manuscript:

$$b_{cell} - b_{est} = ln\left(\frac{\mu_{02,est}}{\mu_{01,est}}\right) \Big/ ln\left(\frac{t_{tot,2}}{t_{tot,1}}\right) \quad , \qquad \text{(S31)}$$

To obtain the value of b_{cell} that best describes the behavior of a cell population, we plot the average difference $<b_{cell} - b_{est}>$ (Eq. S31) as a function of b_{est} for a population of cells and find the value of b where the mean difference is zero.

Section S6. Estimated cytoplasmic extension λ_{init} for the initial rapid entry.

To estimate the magnitude of the material extension during the initial entry phase, we use an approximate geometric model (Figure S3). We make the assumption that the volume to the right of the material element in the undeformed sphere is equal to the volume of material in the pipette:

$$\pi R_p{}^2 L_{init} - \frac{\pi R_p{}^3}{3} = \frac{\pi z_0}{6}(3r_o{}^2 + z_0{}^2) \qquad \text{(S32)}$$

where the dimensions are defied in Figure S3. The volume of the material element is also conserved, requiring that :

$$\pi R_p{}^2 dz = \pi r_o{}^2 dz_0 \qquad \text{(S33)}$$

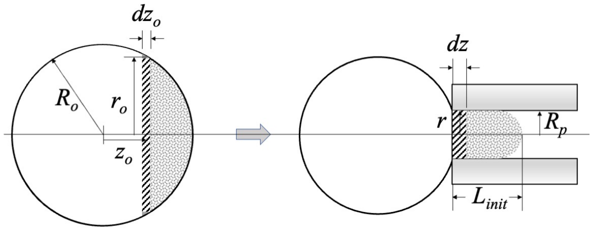

Figure S3. Schematic showing the dimensions of a material element in the undeformed state (left) and the same element at the mouth of the pipette (right). The element is a disc of radius r_o and width dz_o at the position z_o in the undeformed state, and after deformation it is a disk radius R_p and length dz.

We wish to find the axial extension at the mouth of the pipette:

$$\lambda_{init} = \frac{dz}{dz_o} = \frac{r_o{}^2}{R_p{}^2} \qquad \text{(S34)}$$

The radius r_o is related to the axial position z_o by the Pythagorean theorem:

$$r_o{}^2 = 2R_o z_o - z_0{}^2 \qquad (S35)$$

Solving Eqs. S32 and S35 we obtain the following equation for z_o.

$$\frac{z_0{}^3}{3} - R_o z_0{}^2 + R_p{}^2\left(L_{init} - \frac{R_p}{3}\right) = 0 \ . \qquad (S36)$$

The initial projection length L_{init} was obtained by least squares regression of the data as described in section S4. The initial cell radius R_o was determined from the volume of the cell determined from the pipette radius and the fully extended length of the cell (L_{tot}) when it was fully inside the micropipette.

$$V_{cell} = \pi R_p{}^2 L_{tot} - 2\pi R_p{}^3/3 \qquad \text{(S37)}$$

$$R_o = \left(\frac{3}{4\pi} V_{cell}\right)^{\frac{1}{3}} \qquad \text{(S38)}$$

Using MatLab to find the roots of Eq. S38, typically three real roots were found, but only one on the interval $0 < z_o < R_o$. Knowing z_o, r_o and the extension ratio λ_{init} were calculated using Eqs. S34 and S35.

Section S7. Evaluation of an osmotic mechanism for cell volume change.
Measurement of the volume of spherical cells in light microscopy is subject to significant uncertainty because errors in the measurement of the cell diameter enter as the third power into the calculation of the volume. In the case where the cell is fully aspirated into a cylindrical micropipette, however, more accurate determinations of changes in cell volume can be made because the calculation depends linearly on the measurement of the length of the aspirated cell. (The absolute value of the volume is subject to uncertainty in the measurement of the pipette diameter, but for a cell or cells aspirated into the same micropipette accurate comparisons can be made from measurements of the aspirated cell length.) Comparing the lengths of the same cell aspirated at different pressures revealed a trend that the cell volume decreases with increasing aspiration pressure. We postulated that these decreases were due to a hydrostatically driven shift of water from the cell.

The rationale for this follows the correction for cell volume loss in red blood cells when they are partially aspirated at high pressure into a micropipette [4]. The analysis is based on recognition that the membranes of cells are permeable to water, but impermeable to solutes. Force balance reveals that the pressure inside a micropipette aspirated cell is greater than both the pressure in the suspending medium and the pressure in the lumen of the pipette, tending to drive water from the cell. As the water leaves the cell, but solutes remain, an increase in solute concentration tends to pull water into the cell. The flux is predicted to be driven by the sum of these two competing driving potentials [4]:

$$\dot{m}_w = h_P A\left[(p_c - p_o) + \frac{RT}{v_w} ln\left(\frac{1-\chi_{sc}}{1-\chi_{so}}\right)\right] \tag{S39}$$

where $\dot{m}_w$ is the water flux out of the cell, h_p is the hydraulic permeability, A is the area across which transport occurs, p_c is the pressure inside the cell, p_o is the pressure outside the cell (either in the suspending phase or in the pipette), v_w is the partial molar volume of water, RT is the gas constant times temperature, and χ_{sc} is the mole fraction of solute inside the cell. (Note that the product h_pA is known as the filtration coefficient.) Steady state is reached when the flux out of the cell into the pipette lumen is equal to the flux into the cell at the boundary outside the pipette such that the net flux is zero. Assuming that the hydraulic permeability is uniform over the cell surface:

$$0 = \left((p_c - p_o) + \frac{RT}{v_w} ln\left(\frac{1-\chi_{sc}}{1-\chi_{so}}\right)\right)A_o + \left((p_c - p_p) + \frac{RT}{v_w} ln\left(\frac{1-\chi_{sc}}{1-\chi_{so}}\right)\right)A_p \tag{S40}$$

where p_p is the pressure inside the pipette, A_o is the area outside the pipette, A_p is the area over which water can move inside the pipette, and we assume that the outside concentration in the pipette lumen is the same as in the suspending buffer. Rearranging:

$$\frac{1-\chi_{sc}}{1-\chi_{so}} = exp\left[-\frac{v_w}{RT}\frac{(p_c - p_o)A_o + (p_c - p_p)A_p}{A_o + A_p}\right] \quad . \tag{S42}$$

We introduce volume by recognizing that

$$\chi_{sc} = \frac{n_s v_w}{R_w V} \tag{S43}$$

where n_s is the number of solutes inside the cell and R_w is the fraction of the cell volume that is osmotically active. In the resting state prior to activation, the system is at equilibrium with a small concentration difference between the cell interior and the suspending fluid caused by a pressure difference resulting from the cortical tension:

$$\frac{T_{cort}}{2R_0} = \frac{RT}{v_w} ln\left(\frac{1-\chi_{sc0}}{1-\chi_{so}}\right) \qquad (S44)$$

where χ_{sc0} is the mole fraction of solute inside the cell in its resting state, and χ_{so} is the mole fraction of solute in the external media. We note that χ_{so} is related to the solute concentration in the external medium c_o, a known quantity, and χ_{sc0} is related to the number of solutes inside the cell n_s (a constant) and the resting cell volume. Applying the appropriate substitutions, we arrive at:

$$\frac{1}{V_0} = \frac{R_w}{n_s v_w}\left[1-(1-c_o v_w)exp\left(-\frac{v_w}{RT}\frac{T_{cort}}{2R_0}\right)\right] \qquad (S45)$$

By similar reasoning we can obtain an analogous expression for the cell volume when it is being aspirated:

$$\frac{1}{V} = \frac{R_w}{n_s v_w}\left[1-(1-c_o v_w)exp\left(-\frac{v_w}{RT}\frac{(p_c-p_o)A_o+(p_c-p_p)A_p}{A_o+A_p}\right)\right] \qquad (S46)$$

Taking the ratio:

$$\frac{V}{V_0} = \frac{1-(1-c_o v_w)exp\left(-\frac{v_w}{RT}\frac{T_{cort}}{2R_0}\right)}{1-(1-c_o v_w)exp\left(-\frac{v_w}{RT}\frac{(p_c-p_o)A_o+(p_c-p_p)A_p}{A_o+A_p}\right)} \qquad (S47)$$

The exponential term in the denominator of Eq. S47 can be re-written in terms of the aspiration pressure by incorporating expressions for the area in terms of the pipette radius R_p and the radius of the spherical portion outside the pipette R_s,

$$A_o = 2\pi R_s\left(R_s + \sqrt{{R_s}^2 - {R_p}^2}\right) \quad and \quad A_p = 2\pi {R_p}^2$$

making the approximation that the cortical tension is constant and uniform throughout the aspiration, and applying force-balance relationships at the inner and outer cell boundaries:

$$p_c - p_o = \frac{2T_{cort}}{R_s} \quad and \quad p_c - p_p = \frac{2T_{cort}}{R_p}$$

(Note that these relationships assume a negligible contribution from the cytoskeleton at the cell boundary. As we will see, this assumption appears to lead to inconsistencies between theory and experiment.) After algebraic manipulation, we arrive at an expression for the volume ratio in terms of the aspiration pressure $\Delta p = p_o - p_p$:

$$\frac{V_0}{V} = \frac{1 - C_2 exp\left[-\Delta p \frac{v_w}{RT} f(\tilde{R})\right]}{C_1} \qquad (S48)$$

where

$$C_1 = 1-(1-c_o v_w)exp\left(-\frac{v_w}{RT}\frac{T_{cort}}{2R_0}\right),$$

$$C_2 = (1-c_o v_w),$$

$$\tilde{R} = \frac{R_s}{R_p}$$

and,

$$f(\tilde{R}) = \left(\frac{\tilde{R}}{\tilde{R}-1}\right)\left(\frac{\tilde{R}+\sqrt{\tilde{R}^2-1}+1}{\tilde{R}^2+\tilde{R}\sqrt{\tilde{R}^2-1}+1}\right).$$

The expression in Eq. S48 looks promising. As Δp becomes larger, the numerator becomes larger meaning the volume V becomes smaller. The problem comes when realistic values are inserted for the parameters. The partial molar volume of water is approximately 18 liters/mole, the gas constant times temperature is approximately 2.45x10^3 J/mol and aspiration pressures are less than 1,500 Pa. This makes the exponent on the order of 0.01 x $f(\tilde{R})$. When the exponent is small, the exponential term can be approximated by a series:

$$\frac{V_0}{V} = \frac{1}{C_1}\left[1 - C_2\left(1 - \Delta p\frac{v_w}{RT}f(\tilde{R}) + \frac{1}{2}\left(\Delta p\frac{v_w}{RT}f(\tilde{R})\right)^2 + \cdots\right)\right] \qquad (S49)$$

To evaluate the range in which nonlinear behavior can be neglected we must consider the magnitude of $f(\tilde{R})$ in relation to its coefficient. As shown in Figure S4, the function becomes singular as $\tilde{R}$ approaches 1.0, but even at the largest pressures, the squared term in the series expansion is less than 10% of the first term as long as R_s is at least 5.0% larger than R_p. In other words, for the preponderance of every cell aspiration, the relationship between the volume ratio and pressure is expected to be approximately linear according to this model. The actual behavior is clearly non-linear (see Figure 7 of the manuscript), indicating that neglecting cytoskeletal stress contributions at the cell boundary is not proper.

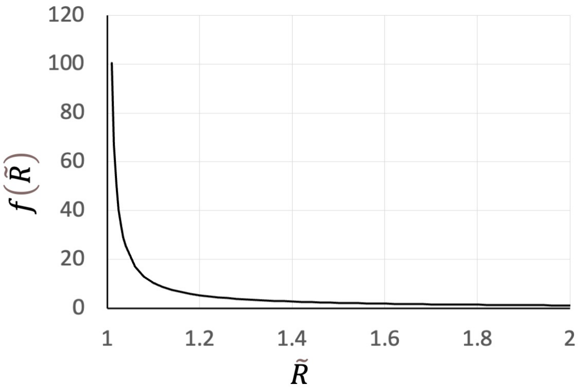

Figure S4. The function $f(\tilde{R})$ as a function of the dimensionless $\tilde{R} = R_s/R_p$.

Section S8. Distributions of properties.

Cell Volume

A

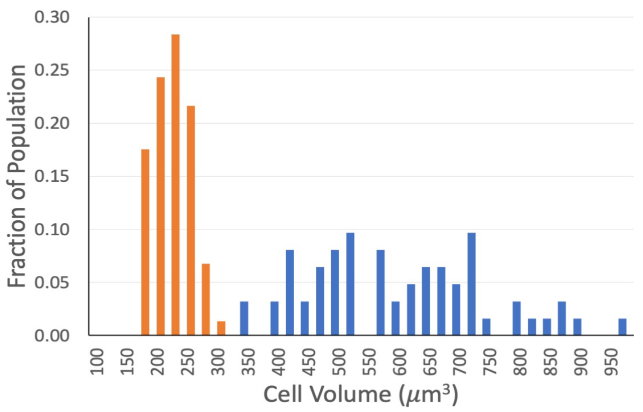

B

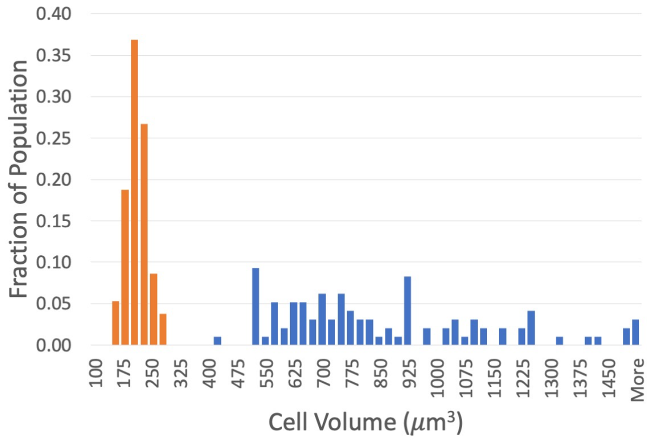

Figure S5. Distribution of cell volumes. A. T cells before (orange) and after (blue) activation. B. CD8+ cells before (orange) and after (blue) activation. Distributions were treated a normal. Activated cells had significantly larger volumes than naïve cells.

Cortical Tension

A

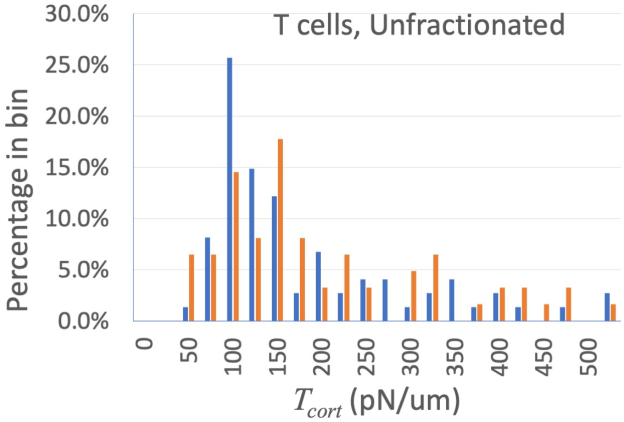

B

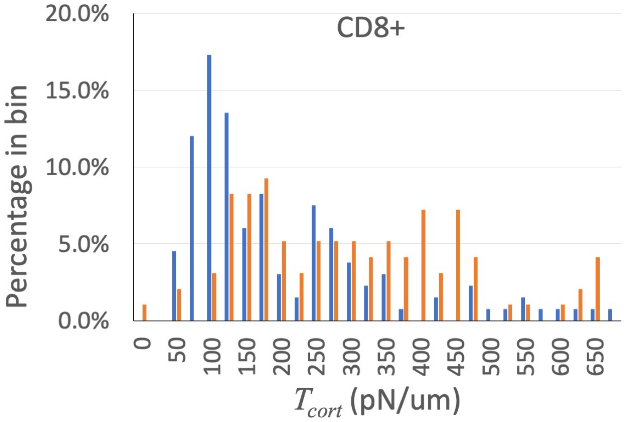

Figure S6. Distribution of cortical tension values for naïve (blue bars) and activated (orange bars) cells. **A.** All T cells. **B.** CD8+ T cells. Only activated CD8+ cells were statistically different from other groups.

Initial projection and its dependence on pressure

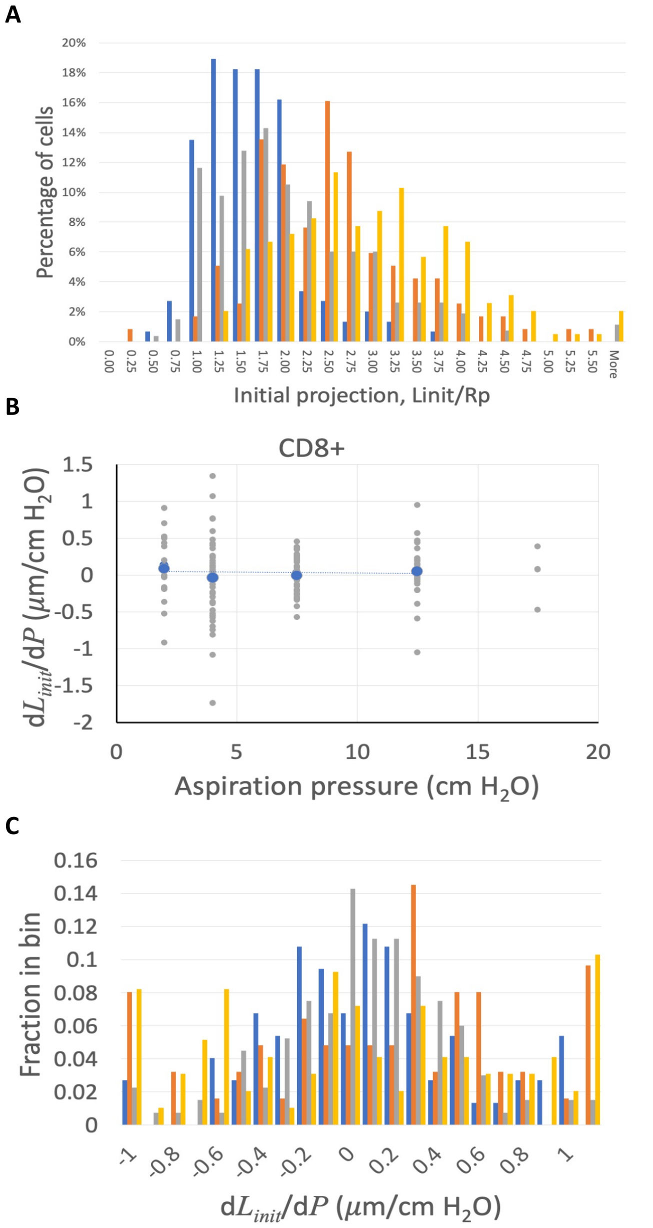

Figure S7. **A.** Initial projection length is normally distributed. Blue: Naïve T cells; Orange: Activated T cells; Gray: CD8+ cells; Yellow: Activated CD8+ cells. Initial projection lengths for activated cells were significantly larger than their naïve counterparts. **B.** Unlike cell volume, the change in L_{init} with pressure does not change with pressure. Results shown for CD8+ cells. The other populations show similar lack of dependence. **C.** The change in L_{init} with pressure is normally distributed with a mean near zero for all cell populations. Color code as in panel A.

Viscosity and Shear Thinning Coefficient

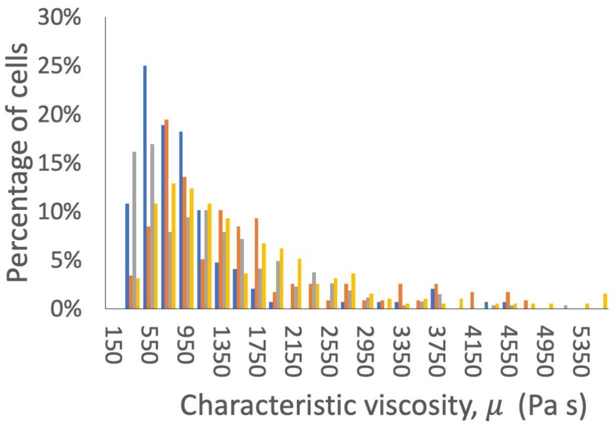

Figure S8. Distribution of characteristic cell viscosities is approximately lognormal. Blue: Naïve T cells; Orange: Activated T cells; Gray: CD8+ cells; Yellow: Activated CD8+ cells. Representative viscosities of activated cells were not significantly different from each other, but the representative viscosity of T cells was significantly smaller than CD8+ cells, and the representative viscosity of both naïve populations were smaller than activated cells.

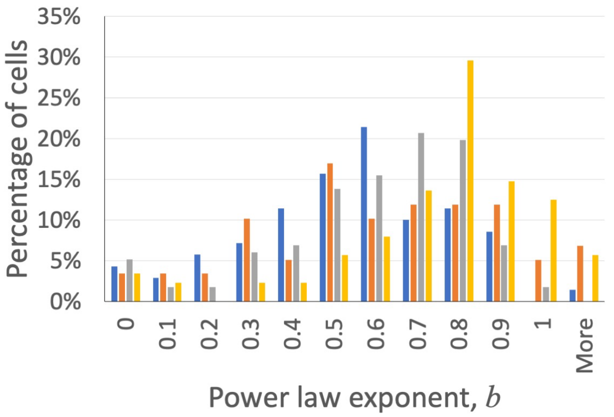

Figure S9. The distribution of the shear thinning coefficient was approximately normal. Blue: Naïve T cells; Orange: Activated T cells; Gray: CD8+ cells; Yellow: Activated CD8+ cells. Only the activated CD8+ cells showed a significant difference from other groups.

References

1. Needham, D. and R.M. Hochmuth, *Rapid flow of passive neutrophils into a 4 microns pipet and measurement of cytoplasmic viscosity.* J Biomech Eng, 1990. **112**(3): p. 269-76.
2. Bird, R.B., W.E. Stewart, and E.N. Lightfoot, *Transport Phenomena*. 1960, New York, NY: John Wiley & Sons, In+c. 780.
3. Malvern, L.E., *Introduction to the Mechanics of a Continuous Medium*. Engineering of the Physical Sciences, ed. J.B. Reswick and W.M. Rohsenow. 1969, Engelwood Cliffs, NJ: Prentice-Hall. 713.
4. Evans, E.A. and R. Waugh, *Osmotic correction to elastic area compressibility measurements on red cell membrane.* Biophysical Journal, 1977. **20**: p. 307-313.
